# Removal of the endothelial surface layer via hyaluronidase does not modulate monocyte and neutrophil interactions with the glomerular endothelium

**DOI:** 10.1111/micc.12823

**Published:** 2023-07-26

**Authors:** ZheHao Tan, Pam Hall, Adam Costin, Simon A. Crawford, Georg Ramm, Connie H. Y. Wong, A. Richard Kitching, Michael J. Hickey

**Affiliations:** ^1^ Centre for Inflammatory Diseases, Monash University Department of Medicine Monash Medical Centre Clayton Victoria Australia; ^2^ Monash Ramaciotti Centre for Cryo‐Electron Microscopy Monash University Clayton Victoria Australia; ^3^ Department of Nephrology Monash Medical Centre Clayton Victoria Australia; ^4^ Department of Pediatric Nephrology Monash Medical Centre Clayton Victoria Australia

**Keywords:** endothelium, glomerulonephritis, glomerulus, hyaluronan, inflammation

## Abstract

**Objective:**

The endothelial surface layer (ESL), a layer of macromolecules on the surface of endothelial cells, can both impede and facilitate leukocyte recruitment. However, its role in monocyte and neutrophil recruitment in glomerular capillaries is unknown.

**Methods:**

We used multiphoton intravital microscopy to examine monocyte and neutrophil behavior in the glomerulus following ESL disruption with hyaluronidase.

**Results:**

Constitutive retention and migration of monocytes and neutrophils within the glomerular microvasculature was unaltered by hyaluronidase. Consistent with this, inhibition of the hyaluronan‐binding molecule CD44 also failed to modulate glomerular trafficking of these immune cells. To investigate the contribution of the ESL during acute inflammation, we induced glomerulonephritis via in situ immune complex deposition. This resulted in increases in glomerular retention of monocytes and neutrophils but did not induce marked reduction in the glomerular ESL. Furthermore, hyaluronidase treatment did not modify the prolonged retention of monocytes and neutrophils in the acutely inflamed glomerular microvasculature.

**Conclusions:**

These observations indicate that, despite evidence that the ESL has the capacity to inhibit leukocyte‐endothelial cell interactions while also containing adhesive ligands for immune cells, neither of these functions modulate trafficking of monocytes and neutrophils in steady‐state or acutely‐inflamed glomeruli.

**Abbreviations**
anti‐GBM Ab: anti‐glomerular basement membrane antibody
ESL: endothelial surface layer
FITC‐WGA: FITC‐wheat germ agglutinin lectin
GN: glomerulonephritis
MP‐IVM: multiphoton intravital microscopy
Ndst1: N‐deacetylase‐N‐sulfotransferase
PE: phycoerythrin
SHAP: serum‐derived hyaluronan‐associated protein
TEM: transmission electron microscopy

## 1 INTRODUCTION

In glomerulonephritis (GN), leukocytes undergo recruitment to the glomerulus where they can play key roles in glomerular injury. Experimentally, in acute forms of GN, neutrophils and monocytes are critical to the response, while in more prolonged forms of GN, T cells and macrophages are prominent.^1^, ^2^, ^3^, ^4^, ^5^, ^6^, ^7^, ^8^ The mechanisms whereby leukocytes are recruited to the glomerulus differ from those in postcapillary venules, where the classical rolling/adhesion/transmigration sequence of leukocyte–endothelial cell interactions underpins leukocyte recruitment. In glomerular capillaries, leukocytes can undergo intravascular arrest, retention and migration in the absence of inflammation, and without an initial rolling interaction.^1^ Furthermore, during acute glomerular inflammation, neutrophils and monocytes undergo prolonged retention within glomerular capillaries from where they promote microvascular injury.^3^, ^4^ Neutrophil retention under these circumstances is predominantly mediated by the β_2_ integrin Mac‐1, while monocytes use LFA‐1, Mac‐1, and the CX3CR1/CX3CL1 pathway.^3^, ^4^ However, no adhesion molecule intervention examined has prevented constitutive neutrophil and monocyte retention in glomerular capillaries.^3^, ^4^ Moreover, the contribution of the endothelial surface layer (ESL), a complex structure on the surface of glomerular endothelial cells, to glomerular leukocyte trafficking is poorly understood.

The ESL is a layer of membrane‐bound and soluble, loosely adherent glycoproteins, proteoglycans and glycosaminoglycans on the endothelial surface.^9^, ^10^, ^11^, ^12^ In glomerular capillaries, the ESL is ~500 nm thick and serves to regulate solute passage through the glomerular filtration barrier.^12^, ^13^, ^14^ The ESL also potentially acts in various ways to impact on leukocyte trafficking.^15^, ^16^, ^17^, ^18^, ^19^, ^20^ Due to its physicochemical properties, it can act as a physical barrier to leukocyte adhesion. This hypothesis is supported by observations in postcapillary venules that interventions that degrade the ESL promote leukocyte adhesion to the endothelium, in the absence of additional inflammatory stimuli.^15^, ^21^, ^22^, ^23^ Conversely, the composition of the ESL incorporates molecules that can promote leukocyte recruitment. The recruitment‐promoting effects of the ESL can occur directly, via molecules such as hyaluronan that can mediate adhesive interactions with leukocytes,^24^, ^25^ or indirectly via residues that can bind leukocyte‐attracting molecules such as chemokines.^18^, ^26^ Little is known about how these diverse capabilities impact on trafficking of myeloid leukocytes to the glomerulus.

One strategy to investigate this is to enzymatically disrupt the ESL and observe the impact of this disruption on leukocyte trafficking. Enzymes such as heparitinase, hyaluronidase, and neuraminidase, which cleave ESL constituents heparan sulfate, hyaluronan and sialic acid, have been shown to alter leukocyte trafficking in a variety of ways.^15^, ^20^, ^25^, ^27^, ^28^, ^29^, ^30^ Most of these experiments have examined leukocyte trafficking in postcapillary venules, where the conventional sequence of leukocyte–endothelial cell interactions occurs. However, in recent years, we have used MP‐IVM to demonstrate the distinct paradigm of leukocyte trafficking in the glomerulus.^3^, ^4^ The aim of the present study was to use MP‐IVM to investigate the contribution of the ESL to monocyte and neutrophil trafficking in the glomerulus.

## 2 MATERIALS AND METHODS

### 2.1 Mice

C57BL/6 wild‐type mice were obtained from the Monash University Animal Research Platform. *Cx3cr1* ^*gfp/+*^ mice expressing GFP under control of the *Cx3Cr1* promoter on a C57BL/6 background,^31^ were bred in‐house. All mice were housed in specific pathogen‐free conditions and fed standard chow. Male mice were used in all experiments. Experiments were approved by the Monash Medical Centre Animal Ethics Committee “B.”

### 2.2 Antibodies and reagents

Disruption of the glomerular ESL was achieved using hyaluronidase (Type IV‐S powder, Sigma‐Aldrich) (35 U i.v., in 0.9% saline solution).^20^, ^29^, ^32^ 0.9% saline solution was used as control. For in vivo imaging experiments assessing neutrophils, anti‐Ly6G (1A8) conjugated to PE (eBioscience) was used. To assess the role of CD44, the function‐blocking anti‐CD44 antibody, KM81 (Thermo Fisher Scientific) was used, with rat IgG_2a_ (BioXCell) used as control. The vasculature was labeled with either Alexa Fluor 680‐conjugated bovine serum albumin (BSA‐AF680), Qtracker® non‐targeted Quantum Dots‐655 (Thermo Fisher Scientific) or rhodamine dextran. For TEM, the following reagents were used: cationic ferritin (ProSciTech); heparin, in HBSS (Hank's balanced salt solution) (Gibco); Schiff's reagent was purchased from Amber Scientific.

### 2.3 ESL assessment—Electron microscopy

Transmission electron microscopy (TEM) assessment of the glomerular ESL was performed using cationic ferritin.^30^, ^33^ The anesthetized mouse was prepared for renal perfusion 15–30 min after enzyme (or saline) administration, as described.^30^, ^33^ In brief, the aorta was exposed via a laparotomy and the blood supply to the right kidney occluded. The aorta was then catheterized retrogradely, distal to the left renal artery. At the time of perfusion, the upper aorta was occluded, and heparinized (5 U/mL) HBSS infused via the catheter (5 mL, 2 mL/min) to exsanguinate the left kidney. Cationic ferritin was then infused (5 mg in 2 mL 0.9% saline, 2 mL/min). The kidney was then removed, the capsule removed and the cortex divided into 1 mm^3^ pieces for fixation in Karnovsky's fixative.^34^ Samples were postfixed in osmium tetroxide/potassium ferricyanide and processed into Epon–Araldite. One micrometre sections were cut and glomeruli assessed for cationic ferritin labelling using Perl's stain. Thin sections were prepared, stained with lead citrate and uranyl acetate and examined via TEM using either a Hitachi H‐7500 or a JEOL JEM‐1400Plus microscope.

The glomerular ESL, detected in TEM sections on the basis of cationic ferritin deposition, was quantitatively assessed using *ImageJ* software,^35^ as previously described.^33^ In brief, three glomeruli were selected randomly from each kidney. From each glomerulus, three capillary cross sections were imaged at 20000×. Images were analyzed for ferritin distribution examining (i) the endothelial surface, (ii) the region beneath the endothelium (sub‐endothelium), and (iii) the fenestrations. For the endothelial surface and the sub‐endothelium, the percentage of the total length covered by ferritin surface was determined, while ferritin distribution in fenestrations was measured as the percentage of fenestrations containing ferritin.

### 2.4 ESL assessment—in vivo imaging

ESL morphology was also assessed via MP‐IVM, staining the ESL using a fluorochrome‐conjugated lectin, as previously described.^20^, ^36^ In brief, mice with intact kidneys were administered either hyaluronidase or saline intravenously (as control). Subsequently, after 30 min, mice received FITC‐WGA (*Triticum vulgaris*, Merck Sigma‐Aldrich, 2 μg/g i.v.) along with the non‐overlapping plasma marker, rhodamine dextran, and glomeruli were imaged immediately via MP‐IVM, using 800 nm laser excitation. A single *z*‐stack of the superficial component of each detectable glomerulus (2–6 glomeruli per mouse) was acquired using 4× zoom. *z*‐slices with clear capillary cross sections were opened in *FIJI* (*ImageJ*) and capillary cross sections with FITC‐WGA staining on the entire endothelial surface were identified. A line was drawn across the capillary profile encompassing a FITC‐WGA staining peak for each side of the vessel. The FITC intensity of the lines intersecting the vessel was determined using the *Plot Profile* tool and the 50% maximum intensity of the normalized maximal value (shown as “normalised FITC‐WGA signal intensity”) determined. Data were derived from 3 to 5 capillary profiles per glomerulus.

### 2.5 Renal multiphoton intravital microscopy (MP‐IVM)

Glomerular leukocyte trafficking was assessed by MP‐IVM of either intact kidneys or post‐hydronephrotic kidneys as previously described.^3^, ^37^, ^38^ The rationale for utilizing both intact and post‐hydronephrotic kidneys was based on our previous studies of glomerular leukocyte trafficking associated with acute inflammation which have consistently demonstrated comparable results using these two methods.^1^, ^3^, ^4^, ^39^, ^40^ Given these results, in this study, intact and post‐hydronephrotic kidneys were used in parallel to address the experimental aims, with the proviso that comparisons between experimental groups were made only between kidneys prepared in the same manner. The technique used for each of the experiments is denoted in the figure legends. In brief, mice were anesthetized using ketamine/xylazine, and a catheter inserted in the jugular vein for administration of fluorochromes and other agents. To image the intact kidney, the kidney was exteriorized via dorsal incisions in the skin and abdominal wall and immobilized in a heated well in a custom‐built stage. For post‐hydronephrotic kidneys, 12 weeks after unilateral ureteric ligation, the hydronephrotic kidney was exteriorized through a lateral incision, drained of urine and secured over a heated platform. In both cases, kidneys were bathed in normal saline and covered with a coverslip, held in place with vacuum grease. Kidneys were imaged using either a Leica SP5 microscope (Leica Microsystems), equipped with a 20× 1.0 NA objective lens and a MaiTai laser (SpectraPhysics), or an Olympus FVMPE‐RS multiphoton microscope (Olympus), equipped with a 25× 1.05 NA water‐immersion objective and an InSight X3 laser (SpectraPhysics). Experiments were performed at 810–900 nm excitation. Recordings were made for 60 min, recording images every 30 s, collecting images of ~150 μm depth containing at least three glomeruli. To visualize neutrophils, C57BL/6 mice received anti‐Ly6G‐PE (2 μg, i.v.) immediately prior to imaging. Monocytes were visualized using *Cx3cr1* ^*gfp/+*^ mice. In some experiments, anti‐Ly6G was used in *Cx3cr1* ^*gfp/+*^ mice to enable simultaneous visualization of monocytes and neutrophils. The vasculature was labeled with either BSA‐AF680 or QTracker‐655. Recordings were analyzed using *Imaris* software (Bitplane). The number of neutrophils and monocytes recruited to the glomerular vasculature and their duration of retention in the glomerulus (dwell time) were analyzed as previously described.^3^ Data from 3 to 5 glomeruli were analyzed and averaged for each mouse.

### 2.6 Experimental protocol

In experiments examining the effects of hyaluronidase treatment on leukocyte trafficking, imaging commenced ~5 min after enzyme administration. To examine the role of CD44, anti‐CD44 (20 μg)^24^ or corresponding isotype control antibody were injected intravenously after enzyme administration, 5 min prior to imaging. To confirm the function‐blocking capacity of anti‐CD44/KM81, preliminary experiments were performed demonstrating the ability of this antibody to reduce neutrophil adhesion in hepatic sinusoids in during endotoxemia (data not shown), as has been shown previously.^24^

To induce GN, mice received anti‐GBM Ab (15 mg, i.v.).^3^, ^4^ In experiments combining enzyme treatment and inflammation, enzymes were administered 55 min after anti‐GBM Ab and recordings of glomeruli were made 60–120 min after anti‐GBM Ab administration.

### 2.7 Statistical analysis

Data are presented as mean ± SEM. Group sizes ranged from 3 to 8 mice. When data were normally distributed and group sizes were similar, statistical analysis was performed using Student's unpaired two‐tailed *t*‐tests, or one‐way ANOVA with Dunnett's multiple comparisons test when greater than two groups were analyzed. Alternatively, unpaired *t*‐tests with Bonferroni correction or Kruskal–Wallis analyses were performed. All analyses were performed using *Prism* 7.02 (GraphPad Software). *p* < .05 were considered statistically significant.

## 3 RESULTS

### 3.1 Hyaluronidase disrupts the glomerular ESL

**FIGURE 1:**
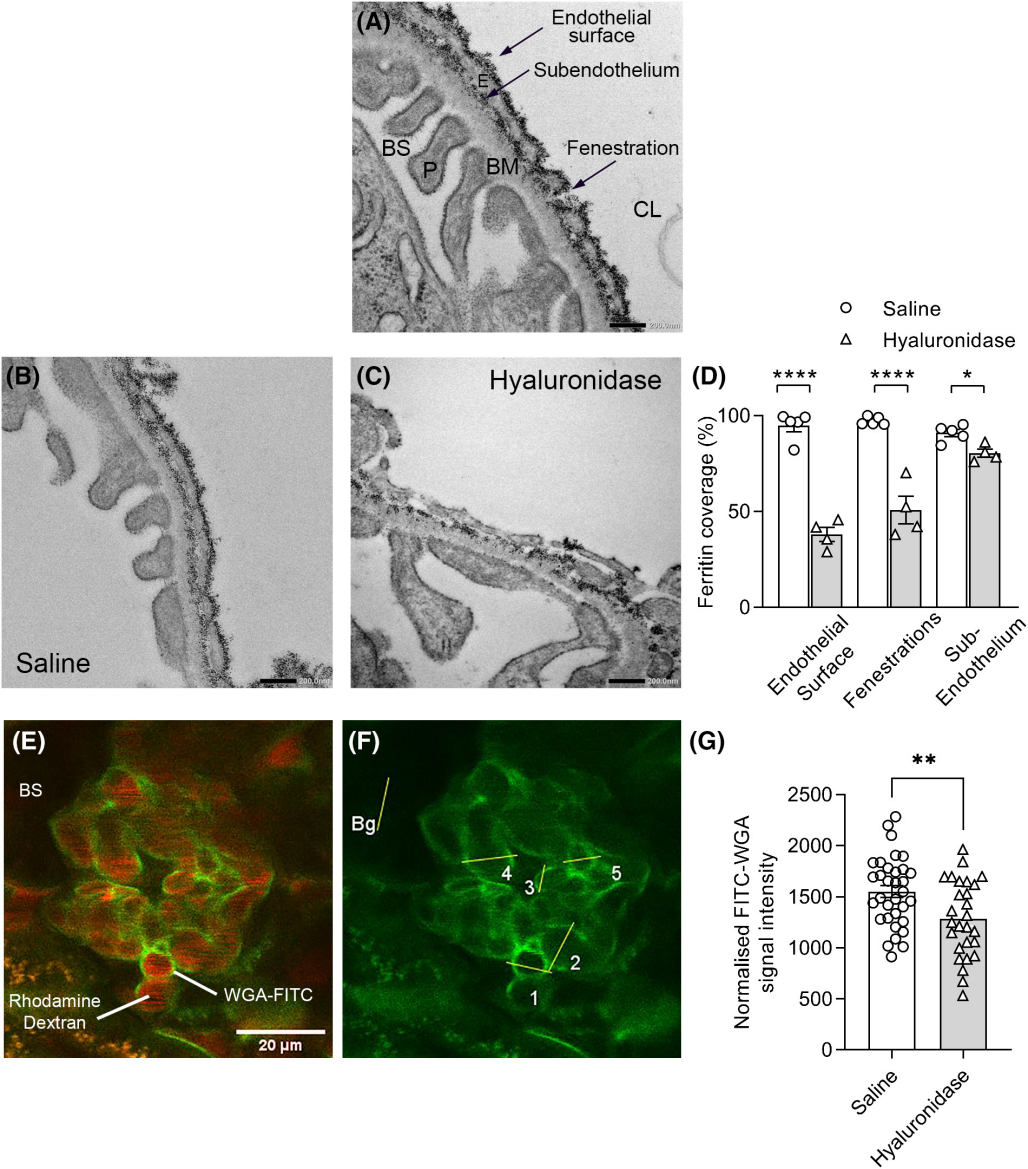
Hyaluronidase disrupts the glomerular endothelial surface layer (ESL). Glomerular ESL structure was assessed in intact kidneys of C57BL/6 mice via either transmission electron microscopy using cationic ferritin, or MP‐IVM imaging of FITC‐WGA staining in glomerular capillaries. (A) Representative electron micrograph of a glomerular capillary wall of an untreated mouse following infusion of cationic ferritin, with the components of the glomerulus labeled: E, endothelium; P, podocyte foot process; BM, basement membrane; BS, Bowman's space; CL, capillary lumen. The three regions of the endothelium assessed for ferritin distribution (endothelial surface, fenestrations, sub‐endothelium) are denoted by arrows. Scale bar‐200 nm. (B, C) Representative micrographs of ferritin distribution in glomerular capillaries after treatment with saline (as control) (B) or hyaluronidase (C). (D) Quantitation of ferritin coverage on the endothelial surface, fenestrations and the sub‐endothelium, showing data for mice treated with saline (control) or hyaluronidase. Data are expressed as a percentage of the total area, or of the number of fenestrations, covered by ferritin staining, and are shown as mean ± SEM of *n* = 3–5 mice/group. (E–G) Assessment of glomerular ESL via staining with FITC‐WGA and in vivo imaging via MP‐IVM. (E) Example single *z*‐plane MP‐IVM image showing ESL staining via FITC‐WGA on the surface of glomerular capillaries (green). The vasculature has been labeled by rhodamine dextran (red). BS, Bowman's space. (F) FITC channel from (E) in isolation, with lines drawn across five capillary profiles denoting locations of quantitative assessment of ESL/FITC‐WGA staining. The location of assessment of background staining in Bowman's space (Bg) is also shown. (G) Assessment of ESL via quantitation of FITC signal intensity at intersections of line with capillary wall, showing data for control (saline) mice, and mice treated with hyaluronidase. Data are shown for 21 or 33 glomeruli for saline and hyaluronidase treatment, respectively, derived from 6 to7 mice/group. *p < .05; **p < .01; ****p < .0001 for comparisons shown, as determined by one‐way ANOVA with Dunnett's multiple comparisons test (D) or Student's *t*‐test (G).

Given the capacity of the ESL component hyaluronan to both contribute to the structural integrity of the ESL and support leukocyte recruitment, we elected to examine the effects of hyaluronidase, which selectively degrades hyaluronan. We first investigated the effects of hyaluronidase on the integrity of the glomerular ESL, using assessment of cationic ferritin binding to examine ESL structure.^24^, ^25^, ^29^, ^30^ Cationic ferritin administered to the circulation binds strongly to the negatively charged ESL and is detectable via TEM.^33^ In normal mice infused with cationic ferritin, and saline‐infused control mice, the glomerular endothelium showed extensive ferritin deposition, including on the endothelial surface, in the sub‐endothelium directly above the glomerular basement membrane, and within the endothelial fenestrations (Figure 1A,B). Quantitation of ferritin deposition revealed that ferritin staining almost completely covered all three regions of the endothelium (Figure 1D). In animals administered cationic ferritin 15–30 min after hyaluronidase (Figure 1C), there was a marked reduction in the amount of ferritin on the glomerular endothelium. This was most evident on the endothelial surface and within the endothelial fenestrations, in which hyaluronidase reduced ferritin deposition by ~50%, compared to saline‐treated control mice (Figure 1D). Hyaluronidase also significantly reduced ferritin deposition in the subendothelium, although this effect was less pronounced (Figure 1D). As an alternate approach for ESL assessment, we applied the previously published approach of using MP‐IVM to investigate ESL staining via FITC‐WGA in glomerular capillaries.^36^ In saline‐treated mice, prominent FITC‐WGA staining was observed lining glomerular capillaries (Figure 1E–G). Hyaluronidase treatment resulted in a significant reduction in the intensity of this staining (Figure 1G), supporting the findings made using TEM. We therefore used hyaluronidase as a tool to assess the contribution of the ESL to glomerular leukocyte trafficking.

### 3.2 Hyaluronidase does not modulate adhesion and retention of monocytes in uninflamed glomerular capillaries

**FIGURE 2:**
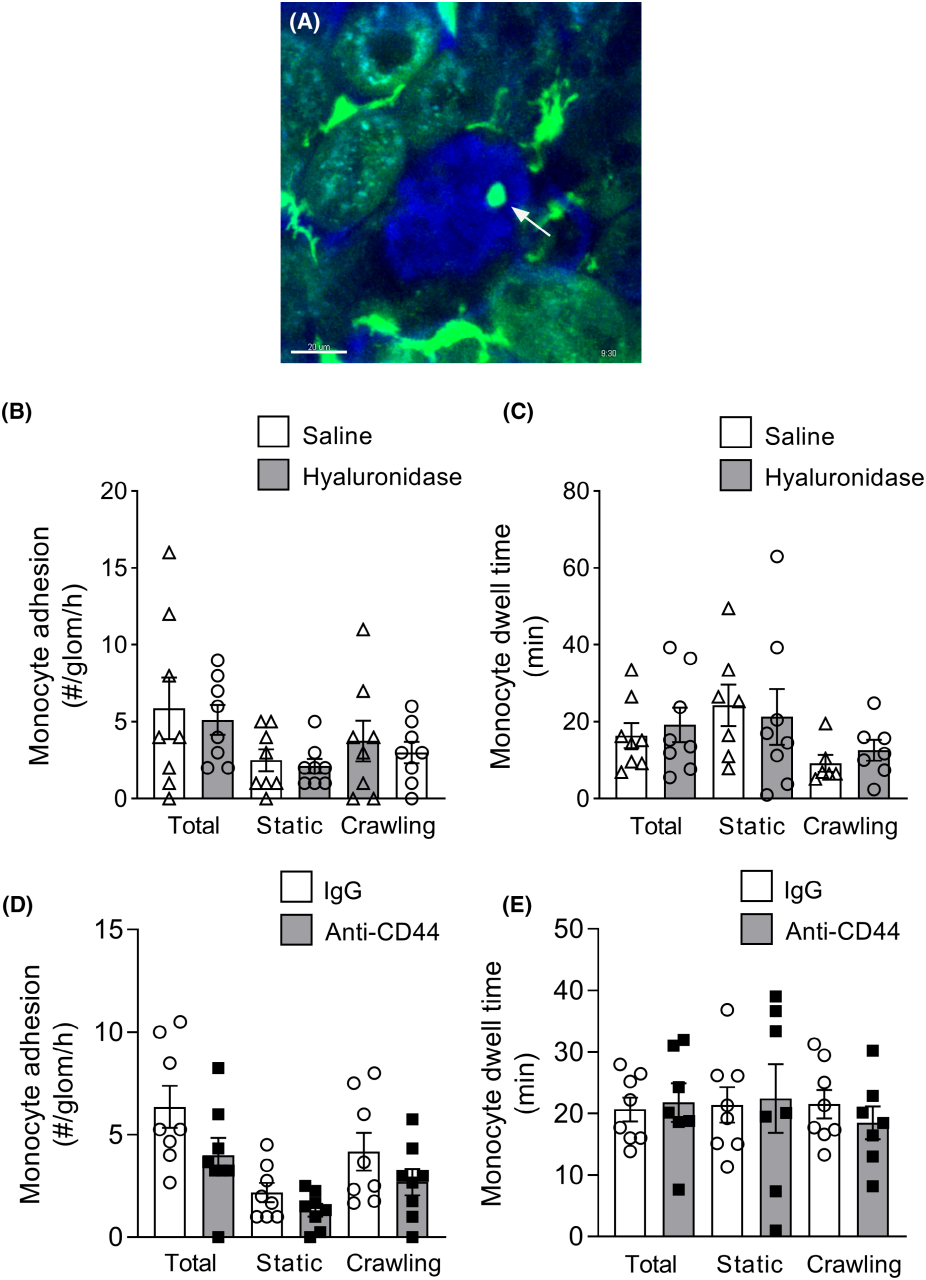
Disruption of the endothelial surface layer with hyaluronidase does not alter adhesion and retention of monocytes within steady‐state glomerular capillaries. (A–C) Recruitment and behavior of monocytes in glomerular capillaries were assessed using MP‐IVM of intact kidneys of *Cx3Cr1* ^*gfp/+*^ mice after treatment with either saline (control) or hyaluronidase. (A) Representative MP‐IVM image of a glomerulus of a *Cx3Cr1* ^*gfp/+*^ mouse (vasculature labeled with BSA‐AF680), containing an intravascular monocyte (arrow), visible via GFP expression. Scale bar‐20 μm. (B, C) Quantitation of monocyte trafficking in glomerular capillaries in mice treated with saline (as control) or hyaluronidase (imaging performed using intact kidneys). Data are shown for the number of monocytes recruited to the glomerulus (expressed as #/glomerulus/h—B), and dwell time of monocytes within the glomerular capillaries (C), shown for total cells, and separately for static and crawling monocytes, for both saline (control) and hyaluronidase‐treated mice. Data are shown as mean ± SEM of *n* = 8 mice/group. (D, E) Effect of anti‐CD44 on monocyte glomerular leukocyte behavior under uninflamed conditions (imaging performed using post‐hydronephrotic kidneys). Number (D) and dwell time (E) of adherent monocytes following treatment of *Cx3Cr1* ^*gfp/+*^ mice with either anti‐CD44 or isotype control antibody, shown for total cells, and separately for static and crawling monocytes. Data are shown as mean ± SEM of *n* = 8 mice/group.

We next examined the effect of hyaluronidase on constitutive glomerular monocyte patrolling, using *Cx3cr1* ^*gfp/+*^ mice to enable detection of monocytes via MP‐IVM (Figure 2A). In control mice, on average ~6 monocytes per hour underwent retention in the glomerulus, with both migratory and static cells observed (Figure 2B). These cells were retained in the glomerular capillaries (dwell time) for ~17 min on average (Figure 2C). To assess the effect of hyaluronidase, mice were treated with the enzyme and after 5 min, underwent a 60‐min period of glomerular imaging. Hyaluronidase had no effect on either the total number of cells undergoing retention in glomerular capillaries or the duration of their retention (Figure 2B,C). These findings demonstrate that significant disruption of the glomerular ESL does not modulate basal glomerular trafficking of monocytes. Furthermore, they provide evidence that hyaluronan within the ESL does not contribute to constitutive monocyte trafficking in glomerular capillaries.

To investigate this pathway further, we also examined the role of the main adhesive ligand for hyaluronan, CD44.^25^, ^41^ CD44 inhibition has been shown to reduce monocyte trafficking in some forms of inflammation indicating that this molecule has the capability of supporting monocyte recruitment.^42^, ^43^ However, CD44 inhibition with the well characterized function‐blocking anti‐CD44 antibody, KM81,^24^ failed to impact either the number or dwell time of adhesive monocytes in the glomerulus (Figure 2D,E). Together these findings indicate that the hyaluronan/CD44 pathway does not contribute to constitutive glomerular trafficking of monocytes under resting conditions.

### 3.3 ESL disruption with hyaluronidase does not modulate adhesion or retention of neutrophils in glomerular capillaries

**FIGURE 3:**
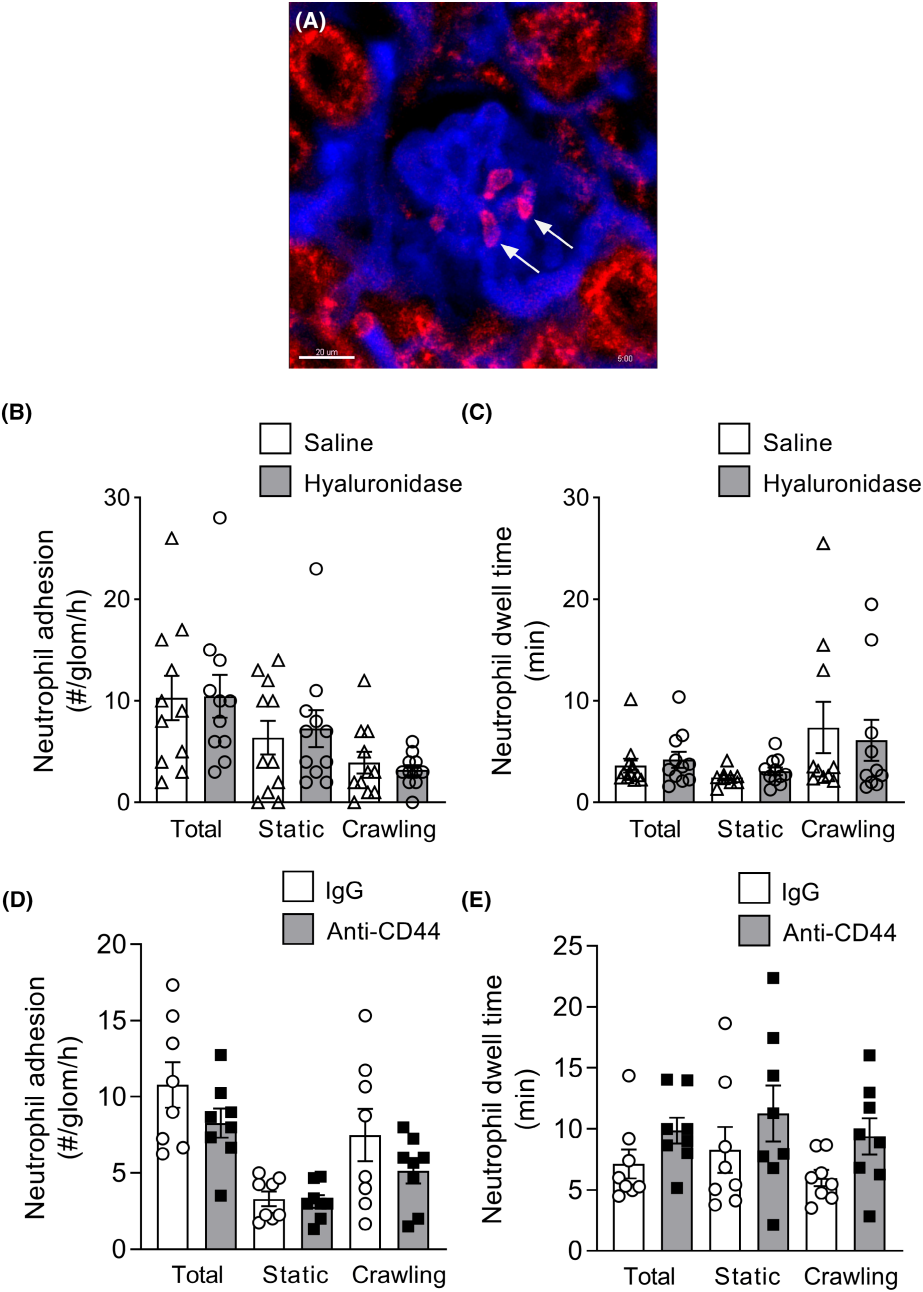
ESL disruption via hyaluronidase does not alter trafficking of neutrophils in glomerular capillaries. (A–C) Neutrophil recruitment and behavior in glomerular capillaries were assessed using MP‐IVM of intact kidneys of wild‐type mice following treatment with either saline (control) or hyaluronidase. (A) Representative MP‐IVM image of a glomerulus of a C57BL/6 mouse (vasculature labeled with BSA‐AF680), containing intravascular neutrophils (arrows), detected via labelling with anti‐Ly6G‐PE. Scale bar‐20 μm. (B, C) Quantitation of neutrophil trafficking in glomerular capillaries in mice treated with saline (as control) or hyaluronidase (imaging performed using intact kidneys). Data are shown for number (B) and dwell time (C) of adherent neutrophils, shown for total neutrophils, and separately for static and crawling neutrophils. Data are shown as mean ± SEM of *n* = 11 mice/group. (D, E) Effect of anti‐CD44 on neutrophil glomerular leukocyte behavior under uninflamed conditions (imaging performed using post‐hydronephrotic kidneys of *Cx3Cr1* ^*gfp/+*^ mice, with neutrophils stained with anti‐Ly6G‐PE). Number (D) and dwell time (E) of adherent neutrophils following treatment with either anti‐CD44 or isotype control antibody, shown for total cells, and separately for static and crawling monocytes. Data are shown as mean ± SEM of *n* = 8 mice/group.

Next, we investigated the effects of ESL disruption via hyaluronidase on neutrophil trafficking, detecting neutrophils in MP‐IVM experiments via in vivo staining with anti‐Ly6G (Figure 3A). In untreated mice, ~10 neutrophils per hour underwent adhesion in glomeruli, with a higher proportion of stationary than migratory neutrophils (Figure 3B), displaying dwell times of ~3.5 min (Figure 3C). Similar to the results seen for monocytes, hyaluronidase treatment did not alter the number or dwell time of adherent neutrophils in glomerular capillaries (Figure 3B,C). As was the case for monocytes, these data demonstrate that disruption of the ESL has minimal impact on glomerular trafficking of neutrophils under steady‐state conditions.

To support these observations, we also examined the role of CD44 in basal neutrophil retention in the glomerulus. CD44 has previously been identified as a ligand of hyaluronan on neutrophils.^24^, ^25^ However, CD44 inhibition did not reduce the number of adherent neutrophils or affect the duration of their retention (Figure 3D,E). Together these findings indicate that the hyaluronan/CD44 pathway is not responsible for constitutive neutrophil adhesion in uninflamed glomerular capillaries.

### 3.4 The glomerular ESL remains predominantly intact during acute immune complex‐induced glomerular inflammation

**FIGURE 4:**
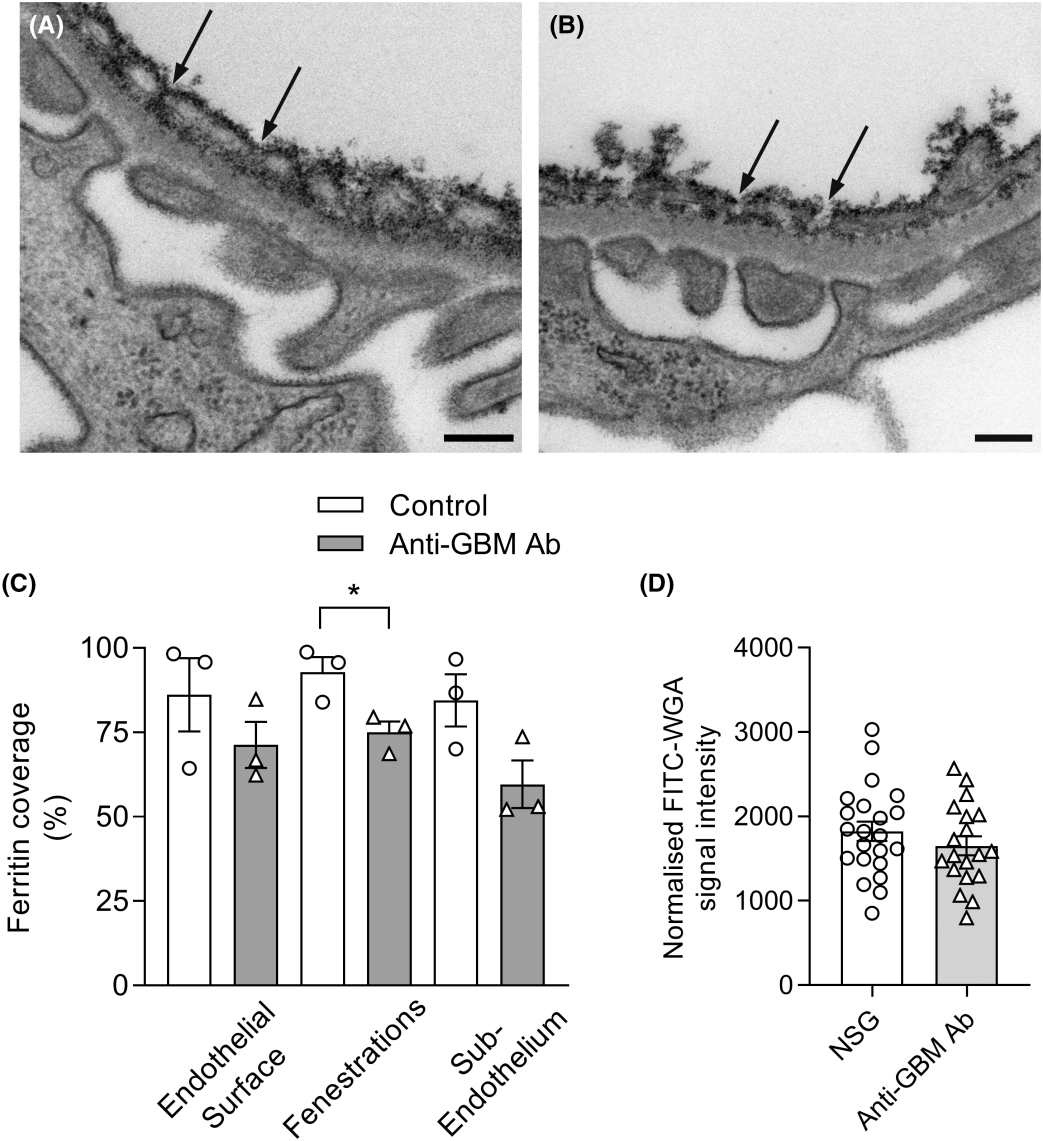
Acute immune complex‐induced inflammation causes only minor disruption to the glomerular endothelial surface layer (ESL). The effect of acute anti‐GBM Ab‐induced glomerulonephritis on glomerular ESL structure was examined in intact kidneys via transmission electron microscopy assessment of cationic ferritin deposition or in vivo staining with FITC‐WGA. (A, B) Representative electron micrographs of glomerular ESL labeled via cationic ferritin 75 min after administration of saline (as control—A), or anti‐GBM Ab (B). Arrows highlight degree of cationic ferritin labelling in endothelial fenestrations under each condition. Scale bars‐200 nm. (c) Quantitation of ferritin coverage for control (saline) mice, and mice 75 min after administration of anti‐GBM Ab. Data show ferritin coverage on the endothelial surface, fenestrations, and the sub‐endothelium, expressed as a percentage of the total area, or of the number of fenestrations, covered by ferritin staining. Data are shown as mean ± SEM of *n* = 3 mice/group. *p < .05 for comparison shown, as determined by unpaired *t*‐test. (D) Assessment of effect of anti‐GBM Ab on ESL via quantitation of FITC signal intensity in mice treated with FITC‐WGA and examined via MP‐IVM. Data are shown for mice treated with either NSG (control) or anti‐GBM Ab. Data are shown for *n* = 27 (NSG) or 24 (anti‐GBM Ab) glomeruli, derived from *n* = 5 or 3 mice/group, respectively.

Inflammation has been shown to modulate the ESL both in the glomerulus and in other vascular locations,^15^, ^21^, ^32^, ^44^ but whether ESL disruption via hyaluronidase would modulate glomerular trafficking of monocytes and neutrophils during acute inflammation was unclear. Therefore, we next investigated the effect of hyaluronidase under acute inflammatory conditions. To address this point, we first examined the effect of acute glomerular inflammation alone on ESL structure, using TEM assessment of cationic ferritin. Mice were treated with anti‐GBM Ab, a stimulus which induces a rapid and significant increase in neutrophil and monocyte retention in glomeruli within 60 min,^3^, ^4^ and glomerular ferritin deposition was assessed after ~75 min (Figure 4). Control (saline‐treated) mice (Figure 4A,C) showed a similar pattern of ferritin deposition to that seen in untreated mice (Figure 1). In anti‐GBM Ab‐treated mice, ferritin staining of the ESL was mostly similar to that in control mice, apart from a minor but significant reduction in the amount of ferritin coverage of the fenestrations (Figure 4B,C). Similarly, in vivo examination of the ESL via MP‐IVM following administration of FITC‐WGA revealed that the ESL was not significantly altered in mice that received anti‐GBM Ab, as compared to NSG‐treated control mice (Figure 4D). Together these experiments revealed that this form of acute glomerular inflammation does not cause major disruption to the ESL.

**FIGURE 5:**
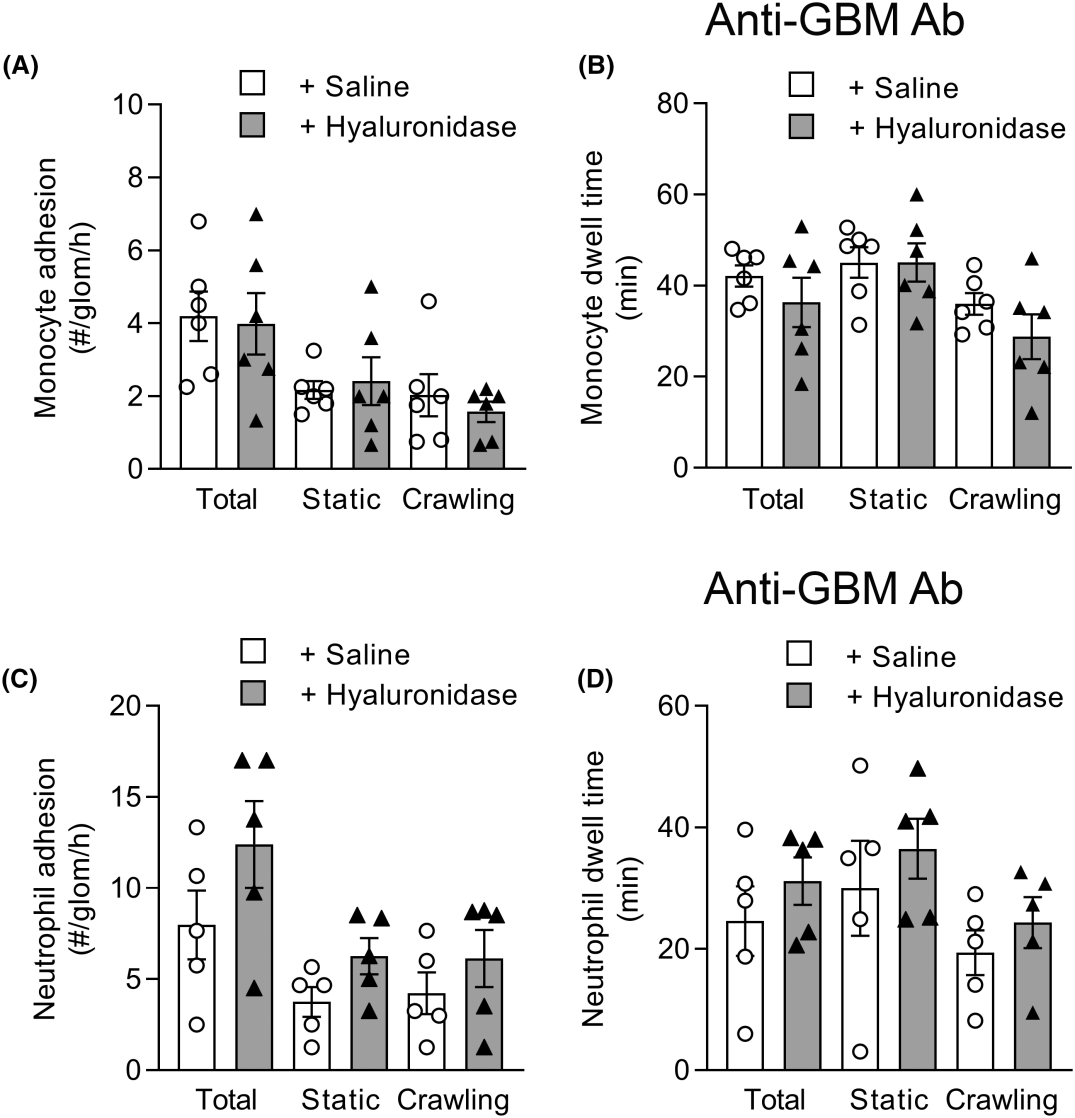
Treatment with hyaluronidase does not modify monocyte or neutrophil trafficking in the acutely‐inflamed glomerulus. Mice underwent the anti‐GBM Ab‐induced model of acute glomerulonephritis followed by ESL disruption via treatment with hyaluronidase, or saline (as control), commencing 60 min after induction of inflammation. Glomerular trafficking of monocytes (A, B) and neutrophils (C, D) were assessed using MP‐IVM in *Cx3cr1* ^*gfp/+*^ mice, or C57BL/6 mice receiving anti‐Ly6G, respectively (imaging performed using post‐hydronephrotic kidneys). Data show number (A, C) and dwell time (B, D) of adherent cells, shown for total cells, and separately for static and crawling cells of each subset. Data are shown as mean ± SEM of *n* = 6 mice/group for *Cx3Cr1* ^*gfp/+*^ mice, or *n* = 4–5 C57BL/6 mice.

Finally, we investigated the effect of ESL disruption on monocyte and neutrophil trafficking under these acute inflammatory conditions. In inflamed mice, monocyte dwell time showed the characteristic increase, to ~40 min, we have described previously.^3^ However, hyaluronidase did not significantly alter the number or dwell time of adherent monocytes under these inflammatory conditions (Figure 5A,B). Similarly, neutrophil dwell time showed the expected elevation in inflamed mice, but hyaluronidase did not alter either the number or dwell time of adherent neutrophils (Figure 5C,D). These findings indicate that disruption of the glomerular ESL makes minimal impact on glomerular trafficking of monocytes and neutrophils during acute inflammation.

## 4 DISCUSSION

Evidence indicates that the glomerular ESL plays an important role in maintaining physiological kidney function, forming the initial element of the glomerular filtration barrier to plasma proteins.^13^, ^14^ In addition to this role, the ESL has the potential to modulate the actions of immune cells within the microvasculature. Numerous studies have demonstrated that disruption of the ESL facilitates leukocyte endothelial interactions, consistent with the hypothesis that the ESL can act as a passive steric barrier to leukocyte recruitment.^15^, ^21^, ^22^, ^23^ Conversely, molecular components of the ESL are capable of supporting the attachment of circulating leukocytes to the endothelial surface.^18^, ^24^, ^25^, ^26^ Very few studies have used direct in vivo visualization of leukocytes in the glomerulus to investigate how these opposing functions play out in the glomerulus. Here we adopted the approach of disrupting the ESL via hyaluronidase and utilizing intravital imaging to examine the effects of this intervention on behavior of monocytes and neutrophils in glomerular capillaries. While hyaluronidase reduced the overall structure of the ESL, thereby reducing its capacity to act as a barrier to leukocyte‐endothelial cell interactions, while also degrading the potential adhesion ligand hyaluronan, it did not lead to any significant changes in intraglomerular trafficking of monocytes or neutrophils. These observations indicate that the potential contributions of the glomerular ESL both as a physical barrier to leukocyte recruitment and as a depot of pro‐adhesive molecules are not active for monocytes and neutrophils under the conditions examined in this study.

Studies of other vascular beds including postcapillary venules in mesentery and skeletal muscle, and pulmonary microvessels, have demonstrated that acute disruption of the ESL is sufficient to increase interactions of circulating leukocytes with the endothelium.^15^, ^21^, ^22^, ^23^, ^44^, ^45^ Given these findings, our observation that disruption of the ESL did not alter constitutive adhesion and migration of monocytes and neutrophils within glomerular capillaries was unexpected. Using two different approaches we confirmed that hyaluronidase was inducing a significant reduction in the ESL indicating that this finding did not stem from a technical failure in hyaluronidase function or delivery. One possible reason for the different observations seen in glomerular capillaries versus those observed in mesentery, muscle, and lung is that trafficking within glomerular capillaries is mechanistically distinct from that which occurs in these other vascular sites. This was demonstrated by our previous observation that neutrophil trafficking to the glomerulus does not follow the conventional rolling/adhesion/transmigration paradigm in that leukocyte arrest can occur without an initial rolling step.^1^ The present observations of a lack of effect of ESL disruption on trafficking of monocytes and neutrophils indicate that the unique nature of leukocyte trafficking in the glomerular microvasculature also applies to the effects of the ESL as a steric barrier to interactions. In contrast to the actions of the ESL in other vascular sites, this role does not apply in the glomerulus, at least for the immune cell populations examined in this study.

We have previously used MP‐IVM to make the unexpected finding that glomerular capillaries support constitutive arrest and intravascular migration of monocytes and neutrophils under steady‐state conditions.^3^, ^4^, ^37^ However the molecular basis of this interaction remains unknown. Inhibition of canonical adhesion molecules has failed to eliminate monocyte and neutrophil trafficking,^3^, ^4^ raising the possibility that a noncanonical adhesion pathway supports these constitutive interactions. Given the capacity of both of these immune cell subsets to undergo CD44‐dependent adhesion via interaction with hyaluronan, it was possible that the CD44/hyaluronan pathway contributed to these interactions in the glomerulus. However, here we demonstrated that neither removal of hyaluronan or inhibition of CD44 impaired constitutive neutrophil and monocyte interactions in glomerular capillaries, indicating that this pathway is not responsible for the attachment of these immune cells to resting glomerular endothelium. Whether these initial interactions with the endothelium are merely the passive consequence of these cells passing through narrow vessels remains unknown. However, it is notable that unbiased analyses of neutrophil adhesion mechanisms in capillary‐rich microvascular beds of the lung, liver, and renal interstitium identified dipeptidase‐1 as a novel, noncanonical adhesion molecule active in capillaries.^46^, ^47^ Whether this is also the case in the glomerulus is yet to be determined. Nevertheless, these latter studies illustrate that there remains potential to identify novel pathways mediating leukocyte adhesion in capillary‐rich vascular beds.

Acute glomerular inflammation results in prolonged retention of monocytes and neutrophils in glomerular capillaries, although the mechanisms underpinning these responses are not fully understood.^3^, ^4^, ^5^ In vascular beds other than the glomerulus, acute inflammatory stimuli such as chemotactic mediators, oxidative stress, and LPS stimulate degradation of the ESL thereby facilitating leukocyte–endothelial cell interactions.^21^, ^22^, ^23^, ^44^ These observations raised the possibility that a similar response would occur in the acutely‐inflamed glomerulus. However, in the present study, inflammation induced by acute in situ immune complex deposition induced only minor changes to the glomerular ESL. Furthermore, the marked changes in leukocyte retention stemming from induction of glomerular inflammation were unaffected by disruption of the ESL via hyaluronidase. These findings indicate that acute inflammation is insufficient to significantly disrupt the glomerular ESL, and that the increased retention of monocytes and neutrophils in the acutely inflamed glomerulus occurs independent of any alteration in the glomerular ESL.

In contrast to our observations in acute glomerular inflammation, studies of chronic kidney disease have demonstrated that the ESL can undergo a range of ultrastructural alterations in disease states. In conditions such as diabetic nephropathy, ischemia/reperfusion and ureteric ligation, the thickness of the ESL in the glomerulus, and the renal interstitial vasculature are reduced.^33^, ^48^, ^49^ In the case of diabetic nephropathy, this alteration can be attenuated by strategies that inhibit inflammation, showing that this response is a consequence of the inflammatory response affecting the kidney.^33^, ^50^ In contrast, in models of lupus nephritis and the genetic disorder Alport syndrome, the ESL undergoes thickening accompanied by glomerular injury and dysfunction.^20^, ^32^ Notably, in the latter studies, increased ESL thickness is associated with induction of hyaluronidase‐sensitive, CD44‐dependent recruitment of activated T cells to the glomerulus, providing clear evidence that the ESL can support recruitment of disease‐promoting leukocytes. While we did not observe a role for this pathway in monocyte or neutrophil recruitment in resting or acutely‐inflamed glomeruli, these observations demonstrate that the response of the ESL to distinct disease stimuli can vary. Notably, in these studies, CD44‐dependent glomerular T‐cell recruitment was observed under conditions in which the ESL was increased, but not in healthy mice with normal ESL structure. This observation indicates that some aspect of the structurally‐altered ESL is responsible for the observed T‐cell recruitment. Whether this stems from a change in its molecular composition or merely its increased size is unclear. Nevertheless, it would be instructive to determine whether under the same circumstances, CD44‐dependent recruitment of monocytes and neutrophils also occurs, or whether this capacity to undergo CD44‐dependent adhesion in the glomerulus is restricted to activated T cells.

Previously, a key role for the hyaluronan/CD44 pathway in neutrophil trafficking has been observed in hepatic sinusoids.^24^ One potential explanation for the difference between these findings and the present observations may be disparities in the levels of hyaluronan in the kidney versus the liver. Under steady‐state conditions, there are no clear differences in the level of hyaluronan between the liver and the kidney.^51^ However, the role for hyaluronan/CD44 in the liver is active during endotoxemia, when expression of hyaluronan in hepatic sinusoids is markedly increased.^24^ This raises the possibility that increased expression of hyaluronan is important for its capacity to mediate leukocyte recruitment in capillary‐sized vessels. In the kidney, increased expression of hyaluronan occurs in glomeruli and the tubulointerstitium in severe, rapidly progressive forms of GN, both experimentally and clinically, and in other prolonged models of renal inflammation.^52^, ^53^, ^54^, ^55^, ^56^ Given this, it is conceivable that the ESL is more capable of supporting glomerular leukocyte recruitment in these severe forms of GN, compared to during acute GN. This idea is supported by the protective effects of CD44 inhibition in prolonged models of crescentic GN and lupus nephritis.^20^, ^57^ In addition, endotoxemia induces expression of SHAP (serum‐derived hyaluronan‐associated protein), which binds to hyaluronan and increases its capacity to support neutrophil adhesion, as shown in hepatic sinusoids.^24^, ^58^ Whether this mechanism is functional in the glomerulus is unclear. However, it is possible that the acute and local inflammatory stimulus of anti‐GBM antibody used here is insufficient to induce expression of this protein. Nevertheless, the role of SHAP is worthy of investigation in more severe forms of GN.

Hyaluronidase has been used to reveal a functional contribution of the hyaluronan/CD44 pathway to leukocyte trafficking in a range of organs,^24^, ^25^, ^59^, ^60^ as well as in glomeruli under select circumstances.^20^, ^32^ A potentially critical difference between most of these studies and those examining the glomerulus is that the glomerular leukocyte trafficking impacted by hyaluronidase only occurred during prolonged disease, in which the ESL was structurally altered by the associated pathology.^20^, ^32^ This is in contrast to other locations where the hyaluronan/CD44 pathway can contribute to leukocyte recruitment induced by acute inflammation.^25^, ^60^ Together with the present observations in which acute glomerular neutrophil and monocyte recruitment did not involve the hyaluronan/CD44 pathway, these findings indicate that one possible unique aspect of this pathway in the glomerulus is the requirement for structural alteration of the ESL to support leukocyte recruitment.

The results in the present study conflict with previous analyses of a mouse lacking Ndst1, a key enzyme in the generation of the ESL.^18^ Ndst1 promotes 6‐O‐sulfation of carbohydrate moieties in heparan sulfate, an abundant glycosaminoglycan in the ESL, and this modification supports granulocyte adhesion to glomerular endothelial cells.^16^ In acute experimental GN, endothelial‐restricted deletion of Ndst1 reduced recruitment of neutrophils and monocytes to glomeruli as assessed histologically, and protected glomeruli from inflammatory injury.^18^ The reason for these differences from the present results is unclear, but it is conceivable that it stemmed from differences in detection of neutrophils when using intravital imaging versus conventional histology, or subtle differences in the model of acute glomerular inflammation used. Nevertheless, as hyaluronidase reduces both hyaluronan and heparan sulfate components of the ESL,^61^ it would be expected that the hyaluronidase treatment would cause loss of the heparan sulfate domain responsible for neutrophil and monocyte adhesion. Given that in the present study, hyaluronidase did not lead to a reduction in neutrophil and monocyte recruitment, further experiments will be needed to confirm whether hyaluronidase treatment does result in loss of the critical heparan sulfate domain within the glomerular ESL.

In conclusion, this study revealed that the role of the glomerular ESL in controlling trafficking of monocytes and neutrophils in the glomerulus is different from that observed in other vascular beds in that disruption of the ESL does not impact on the interactions of these leukocytes with the endothelium. This indicates that the role of the ESL as a barrier to leukocyte recruitment observed in other vascular beds does not apply to neutrophils and monocytes in the glomerulus. In addition, while the structure of the ESL can be modulated in various forms of chronic kidney disease, this response is minimal in an acute, immune complex‐mediated form of glomerular inflammation.

### 4.1 Perspectives

Leukocyte recruitment to the glomerulus contributes to inflammation and injury in immune‐mediated GN, although the role of the ESL in this process remains poorly understood. Here we show that under steady‐state conditions and during acute glomerular inflammation, disruption of the ESL has no impact on the trafficking of monocytes and neutrophils to glomerular capillaries. As the ESL can be altered in different ways during inflammatory conditions affecting the kidney, it will be important to undertake further study to determine if this observation holds true in more severe forms of GN, in order to establish if the ESL is a potential therapeutic target in these conditions.

## AUTHOR CONTRIBUTIONS

Figure and manuscript preparation: ZheHao Tan, Pam Hall, and Michael J. Hickey. Conceptualization: ZheHao Tan, Pam Hall, and Michael J. Hickey. Study design: ZheHao Tan, A. Richard Kitching, and Michael J. Hickey. Data collection: ZheHao Tan, Pam Hall, Adam Costin, Simon A. Crawford, Georg Ramm, Connie H. Y. Wong, and Michael J. Hickey. Data analysis: ZheHao Tan, Pam Hall, and Connie H. Y. Wong. Analysis and interpretation of results: ZheHao Tan, Pam Hall, A. Richard Kitching, and Michael J. Hickey. All authors participated in the review and approval of the final manuscript.

## FUNDING INFORMATION

This work was supported by funding from the National Health and Medical Research Council (NHMRC), Australia (Project Grant IDs 1064112 and 1124459 to MJH and ARK; Senior Research Fellowship ID 1042775 to MJH).

## CONFLICT OF INTEREST STATEMENT

The authors have no conflicts of interest to disclose.

## Data Availability

The data from this study will be made available upon request to the corresponding author (michael.hickey@monash.edu).
